# ATF6α contributes to rheumatoid arthritis by inducing inflammatory cytokine production and apoptosis resistance

**DOI:** 10.3389/fimmu.2022.965708

**Published:** 2022-10-10

**Authors:** Luna Ge, Ting Wang, Dandan Shi, Yun Geng, Huancai Fan, Ruojia Zhang, Yuang Zhang, Jianli Zhao, Shufeng Li, Yi Li, Haojun Shi, Guanhua Song, Jihong Pan, Lin Wang, Jinxiang Han

**Affiliations:** ^1^ Department of Rheumatology and Autoimmunology, The First Affiliated Hospital of Shandong First Medical University, Ji’nan, China; ^2^ Biomedical Sciences College & Shandong Medicinal Biotechnology Centre, Shandong First Medical University & Shandong Academy of Medical Sciences, NHC Key Laboratory of Biotechnology Drugs (Shandong Academy of Medical Sciences), Key Lab for Rare & Uncommon Diseases of Shandong Province, Ji’nan, China; ^3^ Shandong First Medical University & Shandong Academy of Medical Sciences, Ji’nan, China; ^4^ Department of Orthopedic Surgery, The First Affiliated Hospital of Shandong First Medical University, Ji’nan, China; ^5^ Department of Orthopedic Surgery, Shandong Provincial Hospital Affiliated to Shandong First Medical University (Shandong Provincial Hospital), Jinan, China; ^6^ The Second Clinical Medical College, Henan University of Chinese Medicine, Zhengzhou, China; ^7^ Institute of Basic Medicine, Shandong First Medical University & Shandong Academy of Medical Sciences, Ji’nan, China

**Keywords:** rheumatoid arthritis, unfolded protein response, ATF6α, BIRC3, inflammation, apoptosis resistance

## Abstract

**Objective:**

The contribution of activating transcription factor 6α (ATF6α) in rheumatoid arthritis (RA) pathogenesis, especially on fibroblast-like synoviocytes (FLSs), has been suggested by its sensitivity to inflammatory stimulus. However, the exact role and therapeutic potential of ATF6α in RA remains to be fully elucidated.

**Methods:**

ATF6α expression was determined in joint tissues and FLS, and gain-of-function and loss-of-function analyses were applied to evaluate the biological roles of ATF6α in RA FLSs. A murine collagen-induced arthritis (CIA) model, combining both gene deletion of ATF6α and treatment with the ATF6α inhibitor Ceapin-A7, was employed. Joint inflammation, tissue destruction, circulating levels of inflammatory cytokines were assessed in CIA mice. Transcriptome sequencing analysis (RNASeq), molecular biology, and biochemical approaches were performed to identify target genes of ATF6α.

**Results:**

ATF6α expression was significantly increased in synovium of RA patients and in synovium of mice subjected to CIA. ATF6α silencing or inhibition repressed RA FLSs viability and cytokine production but induced the apoptosis. CIA-model mice with ATF6α deficiency displayed decreased arthritic progression, leading to profound reductions in clinical and proinflammatory markers in the joints. Pharmacological treatment of mice with Ceapin-A7 reduced arthritis severity in CIA models. RNA-sequencing of wild-type and knockdown of ATF6α in RA FLSs revealed a transcriptional program that promotes inflammation and suppresses apoptosis, and subsequent experiments identified Baculoviral IAP Repeat Containing 3 (BIRC3) as the direct target for ATF6α.

**Conclusion:**

This study highlights the pathogenic role of ATF6α-BIRC3 axis in RA and identifies a novel pathway for new therapies against RA.

## Introduction

Rheumatoid arthritis (RA) is a chronic inflammatory disorder characterized by synovial inflammation and hyperplasia (1). Fibroblast-like synoviocytes (FLS) are a major contributor to synovial tissue hyperplasia and participate in the degradation of bone and cartilage of RA patients (2, 3). Once FLS gained greater ability to resist apoptosis, they adopt a more effective lead with respect to augmenting RA pathogenesis (4, 5). In recent years, some studies have aimed not only to unveil the disease mechanism of RA, but also to discover the mechanisms underlying FLS inflammatory activities that could be therapeutic targets (6, 7).

Activated RA FLS exhibits a more aggressive and destructive phenotype, thought to be due in part to dysregulation of the unfolded protein response (UPR), a homeostatic signaling pathway designed to respond to the accumulation of unfolded proteins in the endoplasmic reticulum (ER) lumen (8). In mammals, the UPR utilizes three types of sensor proteins, protein kinase RNA-like ER kinase (PERK), inositol requiring enzyme-1(IRE1), and activating transcription factor (ATF6), which detect protein-misfolding pressure in the ER and initiate ER-to-nucleus signaling cascades to maintain a balanced protein quality control system (9). Increased expression levels of key ER stress markers, such as glucose-regulated protein of 78 (GRP78), IRE1, the spliced form of X-box-binding protein 1 (XBP1s), ATF6, and eukaryotic translation initiation factor 2a (eIF2a), have been reported in macrophages and synovial tissues of patients with RA (10, 11).

In RA, inflammation and ER stress act together due to the release of specific cytokines by inflammatory cells to induce ER stress (12). Furthermore, ER stress can amplify the inflammatory response, and the pathogenesis of RA may be sustained by the release of autoantigens that play a dual role in inducing UPR and driving the inflammatory process (13). Therefore, ER stress pathways and their components are attractive targets for drug development and for improving treatment options for RA.

ATF6α is a type 2 transmembrane protein, with ATF6α and ATF6β comprising the two subtypes of ATF6 (14). ATF6α or ATF6β single knock-out mice develop normally whereas mice deficient in both ATF6 subtypes exhibit embryonic lethality (15, 16). Upon ER stress, the N-terminal fragment of ATF6, designated pATF6(N), is cleaved from the parent protein, designated pATF6(P), and is transported into the nucleus, where it binds to the cis-acting ER stress response element (ERSE) and UPR element (UPRE) and increases the expression of genes such as GRP78/BiP, GRP94, protein disulphide isomerase (PDI), the C/EBP homologous protein (CHOP), and XBP1 (17, 18). Although ATF6 is thought to be involved in pro-survival signaling during ER stress, its overexpression can drive the apoptotic pathway (19). Previous studies demonstrated that the expression of cleaved ATF6 increased upon TNFα exposure, but this increase was blocked following the inhibition of proteasome activity or autophagy (20). However, the pathological role of ATF6 in RA remains unclear.

To further explore the role of ATF6α in RA, and also the potential for ATF6α as a therapeutic target for RA, we tested the phenotypic changes in RA FLSs and transgenic models with ATF6α deficiency, and we also examined the efficacy of an ATF6α inhibitor in murine collagen-induced arthritis (CIA) models. We herein demonstrate that blocking ATF6α action reduced arthritic inflammation and disrupted synovial apoptotic resistance. Importantly, ATF6α deficiency or inhibition in CIA models ameliorated arthritic progression, indicating the potential of ATF6α as a treatment target in RA. We further showed that ATF6α positively regulated BIRC3 transcription by binding to the promoter region of the BIRC3 gene. Our findings suggest that ATF6α plays a critical role in RA, including disease progression. Targeting ATF6α may be a novel therapeutic strategy for RA.

## Materials and methods

### Patients

RA and osteoarthritis (OA) patients who underwent knee joint replacement surgery were recruited from the First Affiliated Hospital of Shandong First Medical University. RA patients were diagnosed according to the revised American College of Rheumatology (ACR) criteria (21). Patients with trauma, tumors, diabetes and other autoimmune diseases were excluded from this study. The study was approved by the Institutional Ethics Committee of Shandong Medicinal Biotechnology Center and was conducted in accordance with the Declaration of Helsinki. All the participants signed informed consent. Most participants were treated with anti-rheumatic drugs (DMARDs), including methotrexate, and did not use any biologics during treatment. Detailed data on the patients included in this study were provided in **Supplementary Table 1**. Synovial tissues discarded from joint replacement surgery and blood samples were obtained from these patients.

### Cell culture

Primary FLSs were isolated from the synovial tissue of RA and OA patients as described previously (22). Briefly, synovial tissue was divided into tiny pieces and digested with collagenase (Sigma-Aldrich, St. Louis, MO, USA) at 37°C for 6 h, followed by trypsin treatment. The slurry was then filtered using a 70 μM strainer (Corning, NY, USA) to remove debris. The cells were grown in Dulbecco’s modified Eagle’s medium (DMEM, HyClone, Logan, UT, USA), which was supplemented with 15% fetal bovine serum (GIBCO, Grand Island, NY, USA)) and 1% antibiotics. In this experiment, FLSs from passages 4~6 were employed. MEF cells were extracted from 13.5-day-old mouse embryos from WT and ATF6 KO mice (23). MEFs were cultured in DMEM containing 10% FBS and were used within passage 5 for this study. Peripheral blood mononuclear cells (PBMCs) and lymphocytes were obtained from peripheral blood as described previously (24). To generate PBMCs derived macrophages, PBMSs was cultured in RPMI 1640 media containing 10% FBS and 20 ng/mL GM-CSF((Peprotech, Rocky Hill, NJ, USA)) for 5 days.

### Animals

Animal studies were granted by the Institutional Animal Care and Use Committee of Shandong Medicinal Biotechnology Center. Homozygous ATF6-/- mice on a C57BL/6N background were purchased from Cyagen Bioscience (Guangzhou, China) and DBA/1J mice were from the company of Vital River Laboratory Animal Technology (Beijing, China). The mice were maintained in pathogen-free environments and had free access to take food and water. ATF6-/- mice had backcrossed with DBA/1J mice for at least 6 generations to obtain DBA/1J background. WT littermates (WT) were used as controls. 10-week-old male ATF6-/- mice(n=9), WT mice(n=9) and DBA/1J mice(n=36) were used to construct CIA mice model as previously described (25). Briefly, the 1:1 emulsion (200μL) containing 2 mg/mL bovine type II collagen (Chondrex, Redmond, WA, USA) and full Freud’s adjuvant (Chondrex, Redmond, WA, USA) was injected at the base of the tail. 21 days following the first immunization, a booster immunization with a 1:1 emulsion of bovine type II collagen and incomplete Freud’s adjuvant (200μ L) was given. The arthritis score from 0–4 of each limb was assessed every three day as previously described (26).

Overall, the mice involved in the study were randomly divided into five groups (n=9) for treatment effect evaluation: Control (no CIA induction, no treatment), Vehicle (CIA induction, given volume-matched vehicle control), Ceapin-A7 (CIA induction, given Ceapin-A7 treatment, 10 mg/kg), Etanercept (ETC) (CIA induction, given ETC treatment, 2 mg/kg), Ceapin-A7+ETC (CIA induction, given Ceapin-A7 and ETC treatment, 10mg/kg and 2 mg/kg). Vehicle, Ceapin-A7, ETC, or a combination of Ceapin-A7 and ETC was administered intraperitoneally three times per week from days 22 to days 45. In addition, the thickness of the arthritic hind paws was measured daily every three days with microcalipers. The incidence of arthritis in ATF6 KO and WT mice was calculated. From the first immunization to 45 days, the score of 0 was considered as no incidence.

For the purpose of evaluating safety, 45 extra DBA/1J mice were randomly allocated into five groups (n=9). Vehicle, Ceapin-A7(10 mg/kg), ETC (2 mg/kg), or a combination of Ceapin-A7 and ETC was administered intraperitoneally for 14 consecutive days and blood was collected for blood analysis.

### Micro-computed tomography, Hematoxylin-eosin staining and histopathological examination

To analyze the microstructure of the bone joints, Micro-CT (Quantum GX, PerkinElmer, USA) scans were performed on the hind paws and bone parameters were analyzed in the same region of interest (ROI) of the indibidual mice. The scan parameters were set as follows: exposure at 90 kV, 88 mA for 14 minutes, field of view set to 12.8 mm × 12.8 mm, and resolution set to 2 μm. The bone quality parameters analyzed in this study were the number of trabeculae (Tb.N, 1/mm), trabecular thickness (Tb.Th, μm), trabecular mineral density (Tb.BMD, g/cm3), and trabecular separation (Tb.Sp, mm), which were analyzed using the manufacturer’s software Caliper Analyze. To observe the microscopic cellular morphology of the joint, posterior knee joints with synovial tissue were fixed in 4% paraformaldehyde and then decalcified in 10% EDTA for 3 weeks. After paraffin embedding and sectioning, samples were stained with H&E for light microscopic analysis. The quantitative histopathological analysis was done by three pathologists who evaluated independently.

### Quantitative real-time PCR and RNA sequencing

In this study, to perform quantitative qRT-PCR, total RNA was first extracted from cells using TRIzol reagent (Invitrogen, Carlsbad, CA, USA). Total RNA was then reverse transcribed into cDNA using ReverTra Ace qPCR RT kit (Toyobo, Shanghai, China) and finally amplified using Light- Cycler 480 (Roche, Basel, Switzerland) to amplify under the corresponding primers for qRT-PCR. The primers used in this study were all synthesized by the Beijing Genomics Institute (BGI, Beijing, China), and the sequences of the primers are listed in **Supplementary Table 2**. Relative mRNA levels were analyzed using the 2-ΔCycle Threshold (2-ΔCT) method (27). To perform RNA sequencing, total RNA was first extracted and cDNA libraries were constructed, followed by RNA transcriptome sequencing, which was performed by LC-BIO Technologies Co.,Ltd. (Hangzhou, China).

### Small interfering RNA transfection and Lentivirus transfection

Ruibo Co., Ltd. (Guangzhou, China) synthesized the negative control siRNA (NC), siRNA-ATF6α, and siRNA-BIRC3 (**Supplementary Table 3**) and transfected them into RA FLSs using transfection reagent (PolyPlus Transfection, Strasbourg, France) according to the manufacturer’s instructions.

The human ATF6α and BIRC3 gene overexpression construct was generated using the pLent-GFP-Puro-CMV lentiviral vector (VigeneBiology, Shandong, China). Empty lentivirus was used as the control. The lentivirus was used to infect RA FLSs with enhanced infection solution (VigeneBiology, Shandong, China) according to the manufacturer’s protocol.

### CCK-8, 5-ethynyl-2′-deoxyuridine (EdU) proliferation assay and TUNEL assay

FLSs was seeded on 24-well plates and cultured for 72 h under basal or other intervention conditions. 10 µ l CCK-8 working solution was added to each well, and the culture was continued at 37° C for 1 h. Then, the absorbance at 450nm was detected by a microplate reader (SpectraMax, CA, USA). The EdU working solution (Beyotime, Beijing, China) was preheated to 37°C and then added to the cells to be detected. The cells were cultured for another 6 h and then fixed in 4% paraformaldehyde. After permeating the cells with 0.3% Triton X-100, 100μl EdU solution was added to each well and incubated at room temperature for 30 min away from light. The nucleus is stained with DAPI. A confocal laser was used to acquire images, and image J software was used to count the total cells and EdU positive cells. After the Cells were fixed in 4% paraformaldehyde for 30 min, 50 μl of TUNEL detection solution (Beyotime, Beijing, China) was added to each well. Then, the cells were incubated for 60 min away from light. Nuclei were stained with DAPI. A confocal laser was used to acquire images, and image J software was used to count the total cells and TUNEL positive cells.

### ChIP-qPCR

Briefly, RA FLSs was cross-linked with 1% formaldehyde at 37°C for 10min and stopped by addition of 125 mM glycine. Then, samples were lysed in SDS Lysis Buffer containing 1mM PMSF, and sonicated on ice to generate 200–800 bp DNA fragment. 20μL sample was removed as Input for subsequent assays. Chip-grade ATF6α (Abcam, Cambridge, MA, USA) and normal IgG was used for immunoprecipitation. DNA was recovered after phenol–chloroform extraction and precipitation using ethanol. The precipitated DNA was solubilized with sterile water and PCR analysis was performed with specific ChIP primers(**Supplementary Table 4**). Parameters were set to 30 cycles, 94°C for 1 min, 55°C for 1 min, 72°C for 1 min. The amplified PCR products were analyzed by 1% agarose gel and visualized by ethidium bromide staining.

### Immunofluorescence staining

RA FLSs was fixed in 4% paraformaldehyde for 20 min at room temperature. Then, the cells were permeabilized for 10 min in 0.2% blocked with Immunol Staining Blocking Buffer (Beyotime) for 60min at room temperature. Then, the cells were incubated overnight at 4°C with ATF6α primary antibodies(1:200, GTX15457, GeneTex, CA, USA). After rewarming, cells were incubated for 50 min at 37°C with anti-rabbit secondary antibodies Alexa Fluor-594 (1:500, Protech) for 50 min at 37°C. Nuclei were stained with DAPI. The images were acquired on a confocal laser.

### Enzyme-linked immunosorbent assay(ELISA)

Cell supernatants were collected and centrifuged at 2000 rpm for 5 min, and ELISA kits (Multi Sciences (Lianke) Biotech Co., Ltd., Hangzhou, China) were used to detect IL-6, IL-23A, CXCL1, CXCL8, MMP1, MMP8, ICAM1 and VCAM1 according to the manufacturer’s instructions. For blood samples, blood was centrifuged at 2000 rpm for 5 min, and serum was separated for the above cytokine assays.

### Western blotting analysis

Total protein was prepare using lysis buffer containing protease inhibitors. Protein samples were then sequentially quantified by BCA, boiled with the addition of loading buffer, electrophoresed by SDS-PAGE, and transferred to 0.45μm polyvinylidene fluoride (PVDF) membranes (Millipore, Bedford, MA, USA) and blocked with 5% skim milk powder(Millipore). Next, the membranes were incubated with primary antibodies against ATF6-α, ATF6-β, GRP78, CASP3, Cleaved-CASP3, Bcl2, Bax, BIRC2, BIRC3 and GAPDH (CST, Danvers, MA, USA) at 4°C overnight. Subsequently, the membranes were sequentially re-warmed at 37°C, incubated with HRP-conjugated AffiniPure goat anti-rabbit IgG (H+L) (Proteintech, Wuhan, China) and visualized using an enhanced chemiluminescent substrate (ECL) kit (Vazyme, Nanjing, China). Finally, protein bands were quantified using ImageJ software 2.0 (National Institutes of Health, Bethesda, Maryland, USA) and were normalized to the density of the respective GAPDH band.

### Luciferase assay

According to the manufacturer’s instructions (Promega, Madison, WI, USA), the luciferase assay was performed using Dual-Luciferase assay kit. RA FLSs was transiently transfected with the rDNA-promoter luciferase reporter plasmid and BIRC3 promoter plasmids. ATF6α overexpression or knockdown plasmid was co-transfected. Luciferase activity was determined after treatment for 24 h. Relative luciferase activity was defined as the ratio of firefly luciferase/renilla luciferase (F-Luc/R-Luc).

### Blood analysis

Fresh blood was collected from each mouse and immediately mixed with EDTA for routine blood examination. The EDTA anticoagulated blood were analyzed in an automatic hematology analyzer (Nihon Kohden, MEK-6400 BC-2800vet). For blood biochemical analysis, whole blood obtained was clotted at room temperature for 2 hours and then centrifuged (1000 × g, 10 min) to obtain serum. Chemistry Analyzer (Mindray, BS-350E) was used to measure alanine transaminase (ALT), aspartate transaminase (AST), uric acid (UA) and creatinine (CREA).

### Statistical analysis

GraphPad Prism (Version 8.0, La Jolla, CA, USA) was used for statistical analysis. Statistical analyses between two groups were performed using Student’s t test (normal distribution) or the Mann-Whitney U test (when normal distribution was not given). One-way or two-way ANOVA (if normally distributed) or Kruskal-Wallis ANOVA on Ranks was used if more than two groups were analyzed. Correlation analysis were conducted using Pearson’s correlation. P <0.05 was considered statistically significant (*P<0.05).

## Results

### Increased ATF6α expression in FLSs harvested from RA patients

**Figure 1:**
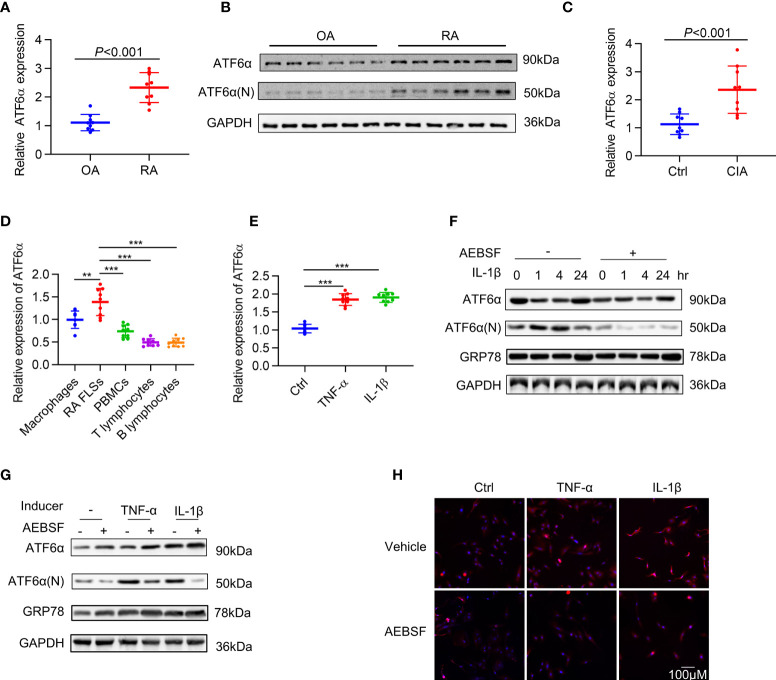
Expression of ATF6α in RA. ATF6α mRNA **(A)** and protein **(B)** expression was assessed in RA FLSs and OA FLSs. **(C)** ATF6α mRNA expression was assessed by qPCR in the knee joints from normal DBA1 mice (Ctrl) and CIA mice. **(D)** ATF6α mRNA expression levels were measured by qPCR in indicated cells from RA patients. **(E)** RA FLSs were stimulated with TNF-α (10 ng/mL) or IL-1β (10 ng/mL) for 24 h and ATF6α mRNA expression was assessed by qPCR. **(F–H)** RA FLSs were pretreated with (+) or without (-) 300 μM AEBSF for 1 h and subsequent TNF-α (10 ng/mL) or IL-1β (10 ng/mL) for the indicated time periods **(F)** or 6h **(G, H)**. **(F, G),** Western blotting was performed to detect ATF6a and GRP78 expression. **(H)**, The cells were then stained with anti-ATF6α antibody (red signal) and DAPI (blue signal). Representative images are shown at 40X magnification. Data were expressed as mean ± SD (*n* = 9), *n* represent biologically independent samples **(A, C–E)**. The data were analyzed using two-tailed unpaired Student’ s *t* test **(A, C)** and one-way ANOVA **(D, E)**. **P < 0.01, ***P < 0.001. ATF6α(N): N-terminal fragment of ATF6α; AEBSF: 4-(2-aminoethyl) benzenesulfonyl fluoride.

To investigate the involvement of ATF6α in RA synovial hyperplasia, its expression was characterized by RT-qPCR and Western blot in FLSs. When compared to FLSs from patients with osteoarthritis (n=9), the expression of ATF6α in RA FLSs (n=9) was markedly increased, both at mRNA (**Figure 1A**) and protein (**Figure 1B**) levels. A comparison of ATF6α mRNA expression in the joints of mice with or without collagen induction revealed that ATF6α was significantly overexpressed in inflamed joints compared to the control joints (P<0.001) (**Figure 1C**). We also examined ATF6α expression in macrophages, PBMCs, T lymphocytes, and B lymphocytes from the peripheral blood of RA patients. ATF6α expression was highest in RA FLSs, followed by macrophages (**Figure 1D**). In contrast to the ATF6α results, we found no significant differences in ATF6β expression in either RA FLSs or inflamed joints from CIA mice compared to their controls (**Supplementary Figure 1**).

To mimic the inflammatory milieu, IL-1β and TNF-α were introduced in the culture medium in RA FLSs to measure the activation of ATF6α. The expression of ATF6α increased significantly in RA FLSs (**Figure 1E**) in response to stimulation by IL-1β or TNF-α. We also examined the activity of ATF6α by analyzing its proteolysis and nuclear translocation. As expected, the proteolysis (**Figures 1F, G**) and nuclear translocation (**Figure 1H**) of ATF6α was triggered by inflammatory factor; however, all these events were blocked when RA FLSs were pre-treated with protease inhibitor, 4-(2-aminoethyl) benzenesulfonyl fluoride (AEBSF), which protects ATF6α against proteolysis. These data indicated that ATF6α can be induced by inflammatory stimuli in RA FLSs and that this induction may be required for its pathological activity.

### ATF6α promotes survival and inflammation in RA FLSs

**Figure 2:**
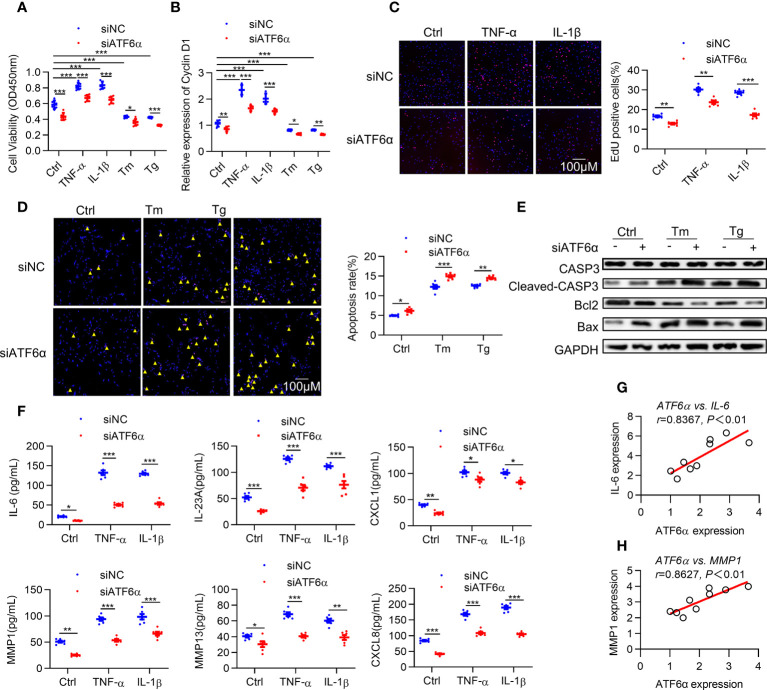
ATF6α is required for the aggressive phenotype of RA FLSs. RA FLSs were transfected with ATF6α-siRNA (siATF6α) or the control (siNC) for 72 h and subsequent vehicle (Ctrl), TNF-α (10 ng/mL), IL-1β (10 ng/mL), Tm (2μg/mL), or Tg (300 nM) for another 72 h **(A)**, 6 h **(B)** or 24h **(C–F)**. **(A)**, Cell viability was detected *via* CCK-8 assay. **(B)** Total cellular RNA was extracted, and cyclin D1 mRNA expression was analyzed by qPCR. **(C)** Cell proliferation was determined by EdU staining, and EdU incorporation was calculated as EdU-positive cells/total cells and quantified by ImageJ. **(D)** Apoptosis was evaluated by TUNEL assay and expressed as percentage of TUNEL-positive cells. **(E)** Total protein was extracted for Western blot detection of CASP3, Cleaved-CAPS3, Bcl2, and Bax. **(F)** Cytokine levels in cell culture supernatants were measured by ELISA. **(G, H)**, Correlations of cytokines (IL-6 and MMP1) and ATF6α expression. Data were expressed as mean ± SD(*n*=6), *n* represent biologically independent samples **(A, B, F)** or fields of view **(C, D)**. The data were analyzed using two-way ANOVA **(A–D, F)** and Spearman correlation analysis. *P < 0.05, **P < 0.01, ***P < 0.001. Tm, tunicamycin; Tg, thapsigargin. The yellow triangle symbol marks TUNEL-positive cells.

Hyper proliferation and apoptotic resistance are key pathological characteristics of RA FLSs that contribute greatly to inflammatory progression and joint destruction (28). Therefore, we investigated the effect of ATF6α on proliferation of RA FLSs. Silencing ATF6α using small interfering RNA (siRNA) inhibited cell proliferation irrespective of the exposure to inflammatory stimuli and ER stress inducers (**Figure 2A**). Similar inhibitory effects on cell proliferation were found when RA FLSs were treated with the ATF6α inhibitor Ceapin-A7 (**Supplementary Figure 2A**).

Cyclin D1 represents an important point for controlling cellular proliferation in response to growth factors (29). Induction of cyclin D1 levels by ER stress was attenuated by knocking down ATF6α or ATF6α inhibitor (**Figure 2B**; **Supplementary Figure 2B**). EdU assay showed that the proportion of cells undergoing proliferation was significantly higher in response to inflammatory stimuli, whereas this increase could be attenuated by silencing or inactivating ATF6α (**Figure 2C**; **Supplementary Figure 2C**). Notably, overexpression of ATF6α promoted the proliferation of OA FLSs (**Supplementary Figures 3A–C**). These results suggest that ATF6α plays an important role in regulating the proliferation of RA FLSs.

Next, we analyzed the impact of ATF6α on apoptosis of RA FLSs. Apoptosis induction resulting from exposure to ER stress inducers [thapsigargin (Tg) and tunicamycin (Tm)] became more obvious when ATF6α was silenced or inactivated in RA FLSs (**Figure 2D**; **Supplementary Figure 2D**), which was also reflected by the increase of Cleaved-caspase3 (CASP3) and B-cell lymphoma-2 associated x protein (Bax) but decrease in B-cell lymphoma-2 (BCL-2) expression (**Figure 2E**). Additionally, the induction of apoptotic death of OA FLSs caused by Tm or Tg treatment could be attenuated by overexpressing ATF6α (**Supplementary Figures 3D, E**).

The above findings suggest that ATF6α plays a key role in maintaining the survival of FLSs in the joints against proapoptotic ER stress conditions. RA FLSs are characterized by an inflammatory phenotype and apoptosis resistance, and whether ATF6α also regulates the inflammatory phenotype of RA FLSs remains unknown. We examined the expression of cytokines interleukin 6 (IL-6), IL-23A, C-X-C motif ligand 1 (CXCL1), CXCL8, matrix metallopeptidase 1 (MMP1) and MMP13, which exacerbate the aggressive phenotype of RA FLSs. The results showed that silencing or inactivating ATF6α could inhibit the expression of these cytokines, whether stimulated by inflammatory factors or not (**Figure 2F**; **Supplementary Figure 2E**). More importantly, the secretion of these inflammatory factors was highly correlated with ATF6α expression (**Figures 2G, H**). The above results suggest that ATF6α is required for the inflammatory phenotype of RA FLSs. In addition, ATF6α overexpression promoted the inflammatory phenotype in OA FLSs (**Supplementary Figure 3F**).

### Decreased arthritis severity in ATF6α-deficient mice

**Figure 3:**
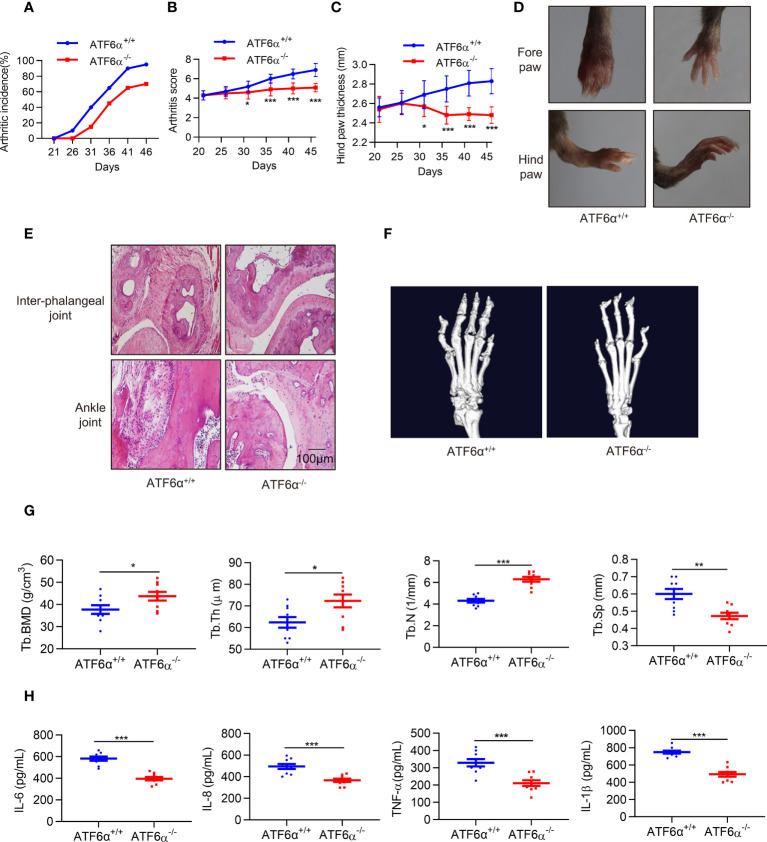
ATF6α deficiency delays CIA development. **(A–C)**, Incidence, arthritis score, and hind paw thickness of CIA in WT and ATF6α KO mice from day 22 to day 45. **(D)**, Representative photographs of the fore and hind paws of CIA in WT and ATF6α KO mice on day 45. **(E)**, Representative H&E staining of inter-phalangeal joints and ankle joints. **(F)**, Micro-CT representative images of hind paws. **(G)**, Quantitative analysis results from micro-CT evaluation. **(H)**, IL-6, IL-8, TNF-α, and IL-1β were measured in mice serum by ELISA. Data were expressed as mean ± SD (*n* = 9) and analyzed using the non-parametric Mann-Whitney test **(B)**, two-way ANOVA analysis **(C)**, and two-tailed unpaired Student’ s *t* test **(G, H)**. *n* represent biologically independent mice (A-C, G, H). *P < 0.05, **P < 0.01, ***P < 0.001. Tb.Th, trabecular thickness; Tb.BMD, trabecular bone mineral density; Tb.N, trabecular number; Tb.Sp, trabecular separation.

Next, we examined the role of ATF6α *in vivo*. CIA models were established in mice with ATF6α deficiency, and the incidence and severity of arthritis were significantly lower in ATF6α-/- mice than in ATF6α wild-type (WT) littermates (**Figures 3A, B**). Paw swelling was also much lower in ATF6α-/- mice than in ATF6α WT mice (**Figures 3C, D**) as assessed by the diameter of the arthritic ankle. Compared with ATF6α WT mice, the ATF6α-/- mice also had reduced inflammatory cell infiltration, joint destruction, and synovial hyperplasia, as evidenced by histological analysis (**Figure 3E**).

Since RA FLSs are involved in bone and cartilage destruction, to further confirm whether ATF6α is involved bone erosion in CIA mice, a micro-computed tomography (micro-CT) scan was performed. ATF6α-/- mice showed a significant improvement in bone erosion, as evidenced by an increase in Tb. BMD, Tb.N, and Tb.Th, but a decrease in Tb.Sp as compared to ATF6α WT mice (**Figures 3F, G**). Moreover, ATF6α insufficiency significantly reduced the secretion of inflammatory factors including IL-6, IL-8, TNF-α and IL-1β in arthritic mice (**Figure 3H**). Collectively, these findings indicate that ablation of the ATF6α effectively prevents synovial proliferation and its destruction of the joints, thereby inhibiting the progression of experimental arthritis.

### Treatment of CIA with an ATF6α inhibitor and/or the TNF-α blocker, etanercept

We first evaluated the toxicity of the ATF6α inhibitor, Ceapin-A7, in DBA1/J mice. When mice were treated by Ceapin-A7 (10 mg/kg) or its vehicle control for 14 consecutive days, no physical or behavioral abnormalities were observed. Moreover, there were no significant changes in both routine blood parameters (**Supplementary Table 5**) and blood biochemistry (**Supplementary Table 6**) parameters compared to the vehicle control.

**Figure 4:**
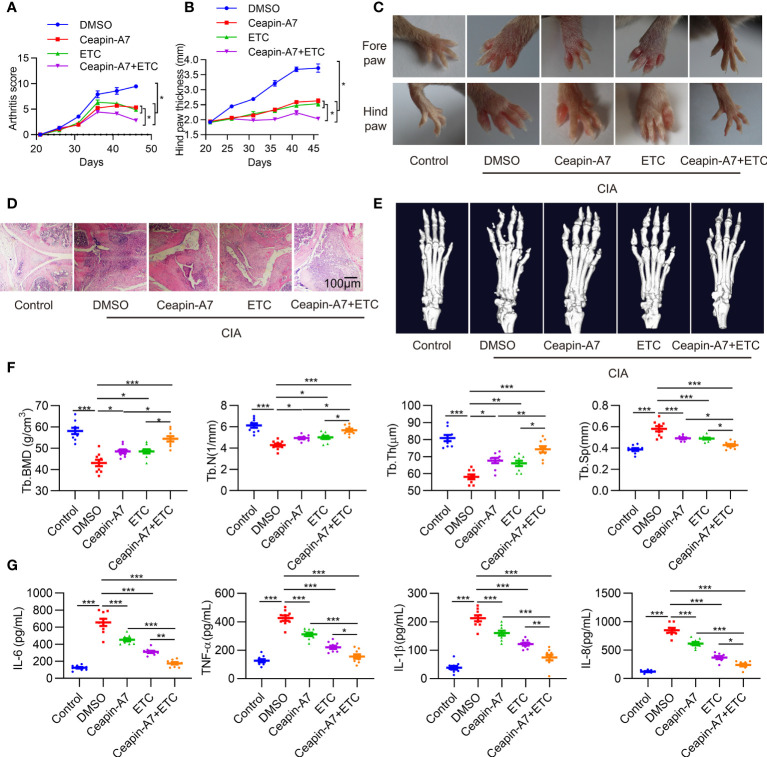
Ceapin-A7 delays CIA development. **(A, B)**, Arthritis score and hind paw thickness of CIA from day 22 to day 45 in indicated groups of CIA mice. **(C)**, Representative photographs of the fore and hind paws of CIA and control mice on day 45. **(D)**, Representative H&E staining of knee joints from CIA mice and the control mice. **(E)**, Micro-CT representative images of hind paws. **(F)**, Quantitative analysis from micro-CT evaluation. **(G)**, Mice serum levels of IL-6, IL-8, TNF-α, and IL-1β were measured by ELISA. Data were expressed as mean ± SD (*n* = 9 in per group), *n* represent biologically independent mice **(A, B, F, G)**. The data were analyzed using two-way ANOVA analysis. *P<0.05, **P<0.01, ***P<0.001. Tb.Th: trabecular thickness; Tb.BMD: trabecular bone mineral density, Tb.N: trabecular number; Tb.Sp: trabecular separation, ETC: etanercept.

To evaluate the anti-arthritic effects of Ceapin-A7, we established the CIA model in DBA/1J mice. Accumulating evidence suggests that TNF-α is a major factor in the pathogenesis of RA (30). Indeed, inactivation of the TNF-α signaling pathway is an effective treatment for RA (31). CIA mice were administered vehicle control, Ceapin-A7 (10 mg/kg), anti-TNF-α (ETC, 2 mg/kg), or a combination of Ceapin-A7 (10 mg/kg) and ETC (2 mg/kg) intraperitoneally three times a week with vehicle. Arthritis scoring was performed every three days from the start of the second immunization, and hind paw thickness was measured and photographed. Ceapin-A7 significantly decreased arthritis scores and the degree of joint swelling, comparable to levels with ETC (**Figures 4A–C**). Furthermore, hematoxylin-eosin (HE) staining showed that the joints in CIA mice underwent obvious synovial hyperplasia, pannus formation, and cartilage destruction when compared to normal joints. Notably, both Ceapin-A7 and ETC ameliorated these pathological changes, and the therapeutic effects were more pronounced when they were combined (**Figure 4D**).

Micro-CT was further applied to evaluate the effect of these interventions on bone erosion. Compared to joints in normal mice, CIA mice showed significant erosion and deformity in both carpal and small toe joints (**Figure 4E**), while intervention with Ceapin-A7 and ETC alleviated bone erosion, as evidenced by the increase of B.BMD, Tb.N, and Tb.Th, but decrease of Tb.Sp (**Figure 4F**). Additionally, inflammatory factors in serum from CIA mice showed significant increases relative to levels in normal mice. Either Ceapin-A7 or ETC could reduce the secretion of these inflammatory factors, and more obvious reduction was found when Ceapin-A7 and ETC were applied together (**Figure 4G**). In addition, qPCR results showed that Ceapin-A7 and ETC had synergistic effects on the expression of IL-6, IL23A, MMP1, MMP13, CXCL1, CXCL8, VCAM1, and ICAM1 in RA FLSs (**Supplementary Figure 4**).

These results suggest that Ceapin-A7 can alleviate arthritis and bone erosion in CIA mice, and its effects are comparable to those of ETC. Notably, a synergistic effect was observed when Ceapin-A7 and ETC were utilized together, suggesting the clinical potential of this combination.

### Identification of BIRC3 as a direct target for ATF6α in RA FLSs activation

**Figure 5:**
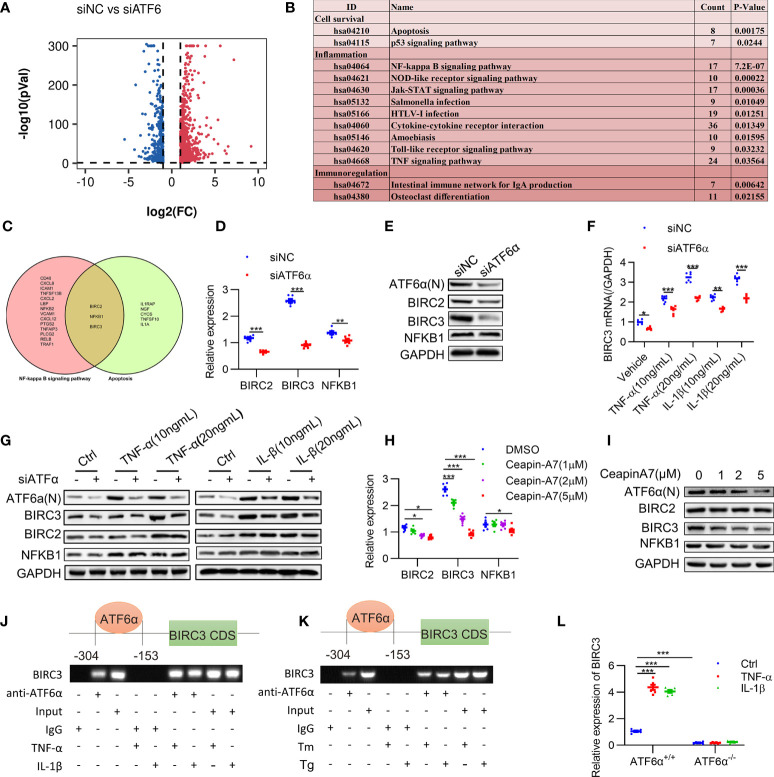
Identification of BIRC3 as the target gene of ATF6α. **(A)**, Volcano plot of differentially expressed genes (DEGs) upon ATF6α knockdown as derived from RNASeq. **(B)**, KEGG enrichment analysis of DEGs was performed, and representative pathways (P<0.05) were listed. **(C)**, Venn diagram of genes of NF-kappa B signaling pathway and apoptosis pathway. **(D–H)**, RA FLSs were transfected with ATF6α-siRNA for 72 h **(D, E)**, followed by treatment with TNF-α or IL-1β for another 24 h **(F, G)**. RA FLSs were treated with ceapin-A7 (0, 1, 2, or 5 μM) for 24 h. BIRC2, BIRC3 or NFKB1 expression was analyzed by qPCR **(D, F, H)** and Western blot **(E, G, I)**. **(J, K)**, RA FLSs were treated with TNF-α (10 ng/mL), IL-1β (10 ng/mL), Tm (2 μg/mL), or Tg (300 nM) for 6 h. ChIP analysis was performed. **(L)** MEF cells isolated from ATF6α+/+ and ATF6α-/- mice were treated with the vehicle (Ctrl), TNF-α (10 ng/mL), or IL-1β (10 ng/mL) for 24 h. BIRC3 expression was measured by qPCR. Data were expressed as mean ± SD (*n* = 6) and analyzed using two-way ANOVA. *n* represent biologically independent samples **(D, F, H, L)**. *P<0.05, **P<0.01, ***P<0.001. ATF6α(N): N-terminal fragment of ATF6α; Tm: tunicamycin; Tg: thapsigargin.

To further elucidate the pathological role of ATF6α in RA, the target genes of ATF6α in RA FLSs were identified. Using RNA sequencing, we examined the global transcriptional response of RA FLSs transfected with siRNA targeting ATF6α or control siRNA. Of the 12,138 genes analyzed, 1407 differentially expressed genes (DEGs) were identified and plotted in a volcano plot (**Figure 5A**), with up-regulated genes displayed in red and down-regulated genes displayed in blue. Of these 1407 DEGs, 133 genes were related to ER stress or the UPR, which is consistent with the biological activity of ATF6α in the UPR. We used qPCR to validate results for several of these DEGs (**Supplementary Figure 5A**).

Previous studies have confirmed that XBP1 and CHOP, which also regulates inflammation and apoptosis, were the direct targets for ATF6α (32, 33). We detected a modest decrease of XBP1 and CHOP expression when ATF6α was silenced in RA FLSs (**Supplementary Figure 5A**). Kyoto Encyclopedia of Genes and Genomes (KEGG) pathway analysis of the DEGs after ATF6α silencing revealed that 13 representative pathways (*P* < 0.05) were significantly associated with cell survival and inflammatory response, including the NF-κB signaling and apoptosis pathways (**Figure 5B**). Comparison of these two pathways revealed three proteins in common, BIRC2, NFKB1 and BIRC3 (**Figure 5C**), suggesting they were potential targets for ATF6α.

Further analysis showed an obvious increase in BIRC3 expression when RA FLSs were treated by TNF-α (10 ng/mL) plus IL-1β (10 ng/mL). However, BIRC2 and NFKB1 were insensitive to TNF-α and IL-1β. Importantly, ATF6α knockdown resulted in more substantial reduction of BIRC3 expression at both mRNA and protein levels when compared to changes in BIRC2 and NFKB1 expression (**Figures 5D–G**). Similar effects were also found when RA FLSs were treated by Ceapin-A7, and furthermore, Ceapin-A7 decreased BIRC3 expression in a dose-dependent manner (**Figures 5H, I**).

To further confirm the likelihood of BIRC3 as a direct target for ATF6α, the chromatin immunoprecipitation (ChIP) assay was performed. PCR amplification revealed the recovery of specific DNA sequences located in the region upstream of the BIRC3 gene, at about 304 to 153 bp upstream from its transcriptional start site. Furthermore, the enrichment of ATF6α on the BIRC3 promoter increased when RA FLSs were challenged by inflammatory stimuli or ER stress inducer (**Figures 5J, K**). Additionally, a luciferase reporter assay showed that overexpression of ATF6α resulted in a marked increase in BIRC3 promoter activity whereas ATF6α knockdown reduced this promoter activity (**Supplementary Figure 5B**). However, the effects of ATF6α on the transcriptional activity of BIRC3 promoter disappeared when t ATF6α were knockout (**Figure 5L**). Taken together, these data support BIRC3 as a direct target of ATF6α in RA FLSs.

### BIRC3 exacerbates the survival and inflammatory phenotype of RA FLSs

**Figure 6:**
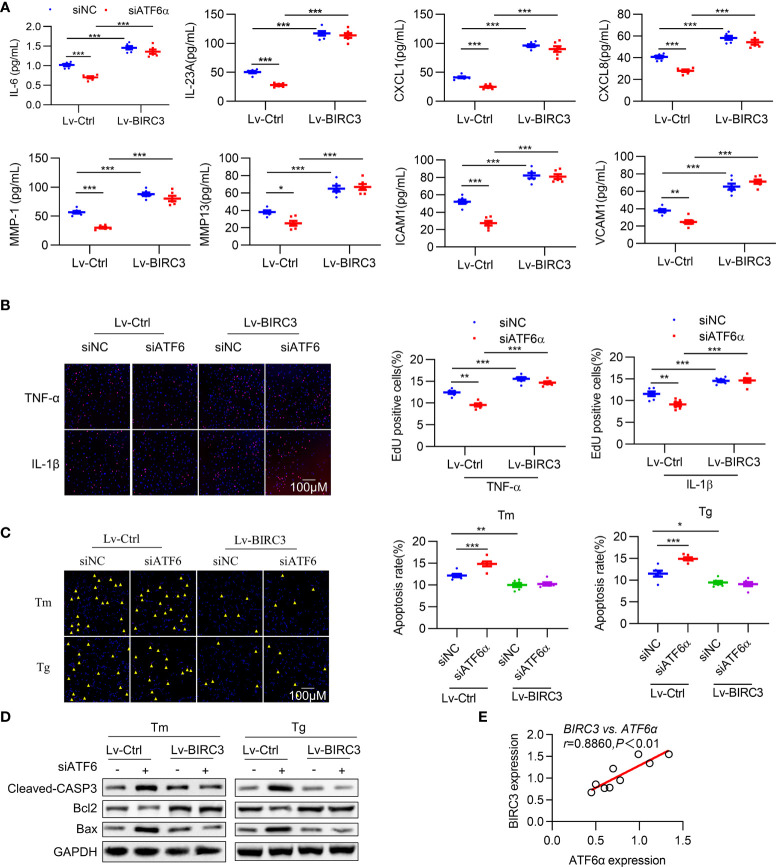
BIRC3 regulates inflammation and apoptosis of RA FLSs. RA FLSs were co-transfected with negative control siRNA (siNC) or ATF6α siRNA (siATF6α) plus empty lentivirus (Lv-Ctrl) or BIRC3-overexpressing lentivirus (Lv-BIRC3) in the presence of Tm or Tg for 72 h. **(A)**, IL-6, IL-23A, ICAM1, VCAM1, CXCL1, CXCL8, MMP1 and MMP13 levels in cell culture supernatants were measured by ELISA. **(B)**, EdU assay was performed after overexpressing BIRC3 in ATF6α-silenced RA FLSs for 72 h in the presence of TNF-α or IL-1β (10 ng/mL). EdU incorporation was calculated as EdU + cells/total cells per field, quantified by Image J. **(C)**, Apoptosis of RA FLSs was detected by TUNEL assay. The data are expressed as the percentage of TUNEL-positive cells per field. **(D)**, Total protein was extracted for Western blot detection of Cleaved-CASP3, Bcl2, and Bax. **(E)**, Correlations of BIRC3 and ATF6α expression. Pearson’s correlation coefficient (r) and P value are shown. Data are mean ± SD(*n*=6) and analyzed using one-way ANOVA **(A–C)** or Pearson correlation analysis **(E)**. *n* represent biologically independent samples **(A)** or fields of view **(B, C)**. *P < 0.05, **P < 0.01, ***P < 0.001. Tm. tunicamycin; Tg, thapsigargin. The yellow triangle symbol marks TUNEL-positive cells.

We further characterized the expression of BIRC3 in RA and determined that BIRC3 expression in RA FLSs was significantly higher at the mRNA and protein levels than in OA FLSs (**Supplementary Figures 6A, B**). Consistently, BIRC3 expression in arthritic joints from CIA mice was also significantly increased when compared to the normal controls (**Supplementary Figure 6C**). Additionally, silencing BIRC3 reduced the secretion of inflammatory factors in RA FLSs in both basal and inflammatory states (**Supplementary Figure 6D**), and BIRC3 overexpression abrogated the mitigating effect of ATF6α knockdown on inflammation (**Figure 6A**).

Multiple studies have demonstrated that BIRC3 is required for maintaining cell survival (34), and our results indicate that the inhibitory effects of ATF6 knockdown on the proliferation of RA FLSs were largely counteracted by BIRC3 overexpression (**Figure 6B**). To test whether BIRC3 can change the apoptotic properties of RA FLSs, we next overexpressed BIRC3 in RA FLSs when ATF6α was silenced. Knockdown of ATF6α increased apoptosis in RA FLSs under Tm or Tg stimulation, but this increase could be attenuated by BIRC3 overexpression (**Figure 6C**). Further detection of apoptosis-associated proteins further confirmed that BIRC3 mediated the anti-apoptotic effect of ATF6α (**Figure 6D**). Moreover, a significant positive correlation was observed between BIRC3 and ATF6α expression in RA FLSs (**Figure 6E**). Collectively, these results show that BIRC3 is critical for ATF6α-induced pro-inflammatory responses and apoptosis resistance in RA FLSs.

## Discussion

This study elucidated the pathogenic mechanisms and therapeutic potential of ATF6α in RA. According to the data obtained *in vitro*, ATF6α promotes the expression of key inflammatory factors and reduces cellular apoptosis in RA FLSs. In addition, we used a mouse CIA model lacking the ATF6α gene to determine the role of ATF6α in CIA. We measured multiple parameters (i.e., clinical arthritis scores, joint tissue inflammation/destruction, serum levels of proinflammatory cytokines) *in vivo* experiments, and the results clearly showed that ATF6α is required for the CIA progression, as mice lacking ATF6α were protective against CIA. These evidence support the important role of ATF6α in the development and progression of destructive arthritis.

BIRC3 (cellular IAP2) belongs to the human inhibitors of apoptosis protein (IAP) family (35), is one of the eight members. Previous studies have shown that BIRC3 is a multi-functional protein, as demonstrated by its ability to regulate not only caspases and apoptosis, but also inflammatory signaling, immunity, mitogenic kinase signaling, and cell proliferation (36). In classical TNF-α signaling in RA FLSs, BIRC2/BIRC3 forms a complex with TNFR2 and TRAF2 to further activate the NF-κB and MAPK pathways (37, 38). Although both apoptosis resistance and immune responses contribute to the development and progression of autoimmune diseases such as RA, relatively little is known about the regulation of BIRC3 at the interface of the two phenotypes in RA. Here, we identified BIRC3 as a direct target of ATF6α in RA FLS and further confirmed that ATF6α-BIRC3 serves as an important link between apoptosis resistance and hyperimmune responses, thereby conferring the destructive phenotype of RA FLSs (**Figure 6**).

In autoimmunological diseases, inflammation and the ER stress pathways are complex and may be concurrently regulated. Therefore, in terms of clinical benefit, targeting a single pathway does not appear to lead to greater therapeutic benefit. Instead, a more effective approach to better control the RA disease process should be to modify integrated biological outcomes by simultaneously targeting both ER stress and inflammation pathways. Previous studies demonstrated that ATF6α serves as the target for SubAB to inactivate NF-κB signaling (39). Another important finding of this study is that pharmacologic effect of the ATF6α inhibitor Ceapin-A7 could ameliorate the inflammatory phenotype in RA FLSs and CIA model mice as effectively as TNF-α blockers (40–42) (**Figure 3**). Furthermore, combining anti-TNF therapy with a cIAP1/2 inhibitor in RA patients may have the potential to extend a potent disease-suppressive effect into long-term, treatment-free remission. Likely, the ATF6α inhibitor and TNF-α blocker synergistically suppress the destructive phenotype of RA-FLSs (**Supplementary Figure 4**) and arthritic progression in CIA models (**Figure 3**). It is suggested that ATF6α inhibitors may constitute a new class of anti-rheumatic drugs that act conjunction with biological therapies targeting immunity.

The present study also has limitations, including: i) a focus on the mechanism of ATF6α-mediated activation and maintenance of RA-FLSs, which does not exclude the possibility that ATF6α may also promote arthritic progression by influencing other immune cell functions (e.g., T cells, macrophages, and regulatory T cells; ii) no profiling of targeted genes through ChIP-sequencing, which warrants further studies; iii) the sample size of the RA patient study was small, so the results may not be fully generalizable.

In summary, our findings revealed a key role for the ATF6α/BIRC3 axis in RA and further demonstrated that ATF6α/BIRC3 bridges the inflammatory and apoptotic-resistance phenotypes in RA FLSs. Additionally, ATF6α deficiency or an ATF6α inhibitor reversed the histologic and molecular signs of inflammation in CIA models, suggesting it may serve as a treatment option for RA.

## Data availability statement

The RNA-sequencing data presented in the study are deposited in the GEO repository, accession number GSE214842.

## Ethics statement

The studies involving human participants were reviewed and approved by Institutional Review Board of Shandong Medicinal Biotechnology Center, Shandong First Medical University (approval ID: FMU191016). The patients/participants provided their written informed consent to participate in this study. The animal study was reviewed and approved by Institutional Animal Care and Use Committee of Shandong Medicinal Biotechnology Center (SMBC-19-003).

## Author contributions

All authors were involved in drafting the article or revising it critically for important intellectual content, and all authors approved the final version to be published. JH had full access to all the data in the study and takes responsibility for the integrity of the data and the accuracy of the data analysis. Study conception and design: JH and LW. Acquisition of data: LG, TW, DS, YG, HF, RZ, and HS. Analysis and interpretation of data: JZ, SL, YL, GS, and JP.

## Funding

This work was supported by National Natural Science Foundation of China (Grant No. 81772760, 82072850, 81901666, 82101903, 82171801), The Shandong Taishan Scholarship (Grant NO. tsqn20161076), Natural Science Foundation of Shandong Province (Grant No. ZR2020YQ55), Key Research and Development project of Shandong Province (No. 2021ZDSYS27), The Innovation Project of Shandong Academy of Medical Sciences (2021), The Youth Innovation Technology Plan of Shandong University (Grant No. 2019KJK003) and Academic Promotion Programme of Shandong First Medical University (Grant No. 2019LJ001).

## Acknowledgments

We apologize to colleagues whose work could not be cited due to space constraints.

## Conflict of interest

The authors declare that the research was conducted in the absence of any commercial or financial relationships that could be construed as a potential conflict of interest.

## Publisher’s note

All claims expressed in this article are solely those of the authors and do not necessarily represent those of their affiliated organizations, or those of the publisher, the editors and the reviewers. Any product that may be evaluated in this article, or claim that may be made by its manufacturer, is not guaranteed or endorsed by the publisher.
