# Supplementary material for: ATF6α contributes to rheumatoid arthritis by inducing inflammatory cytokine production and apoptosis resistance
Source: Front Immunol. 2022 Oct 10;13:965708. doi: 10.3389/fimmu.2022.965708 (PMC9590309; doi:10.3389/fimmu.2022.965708)
Supplement: Supplementary file 1 [file DataSheet_1.docx]

Supplementary Material

## Supplementary Figures


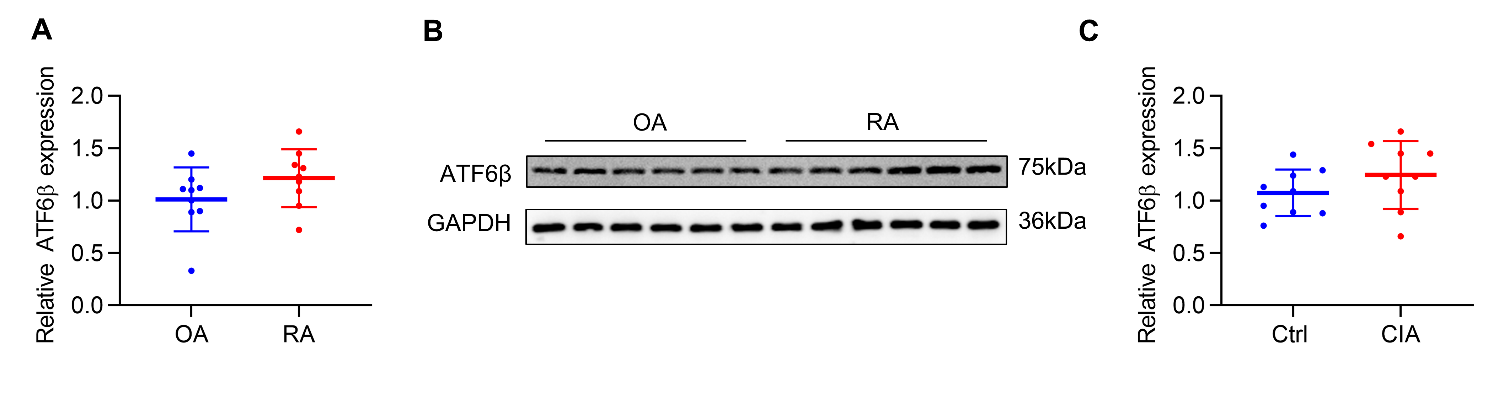
**Supplementary Figure 1.** Expression of ATF6β in RA. **A,** ATF6β mRNA expression was assessed by qPCR in RA FLSs (*n*=9) and OA FLSs (*n*=9). **B,** ATF6β protein expression levels in RA FLSs (*n*=6) and OA FLSs(*n*=6) were assessed by Western blotting. **C,** ATF6β mRNA expression was assessed by qPCR in the knee joints from normal DBA1 mice (Ctrl, *n*=9) and CIA mice (CIA, *n*=9). *n* represent biologically independent samples (A, C). Date was expressed as mean±SD. The data was analyzed using two-tailed unpaired Student’s *t* test (**A, C**).


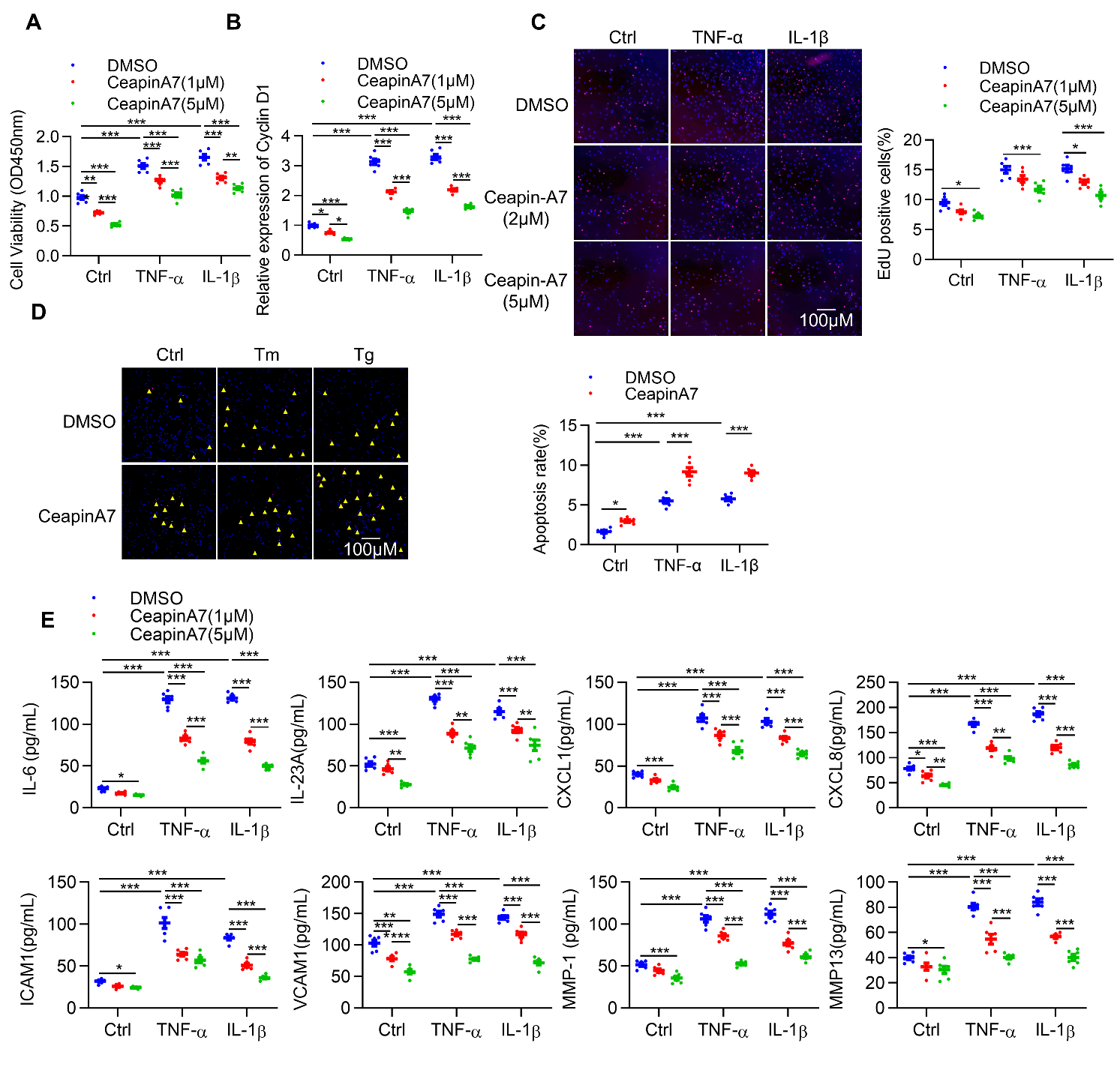


**Supplementary Figure 2**. Ceapin-A7 ameliorates the inflammatory phenotype of RA FLS. RA FLSs was treated with Ceapin-A7(1μM, 5μM) for 72 h**(A)** or 6h**(B)**. **A,** Cell viability was detected via CCK-8 assay. **B,** Total cellular RNA was extracted and cyclin D1 mRNA expression was analyzed by qPCR. **C,** RA FLSs was pretreated with Ceapin-A7(1μM, 5μM) for 2h, followed by TNF-α (10 ng/mL), IL-1β (10 ng/mL) for 24 h. Cell proliferation was determined by EdU staining and EdU incorporation was calculated as EdU + cells/total cells, quantified by ImageJ. **D,** RA FLSs was pretreated with Ceapin-A7(5μM) for 2h, followed by Tm (2μg/mL) or Tg (300 nM) for 24 h. Apoptosis was evaluated by TUNEL assay and expressed as percentage of TUNEL-positive cells. **E,** RA FLSs was pretreated with Ceapin-A7(1μM, 5μM) for 2h, followed by TNF-α (10 ng/mL) or IL-1β (10 ng/mL) for 24 h. IL-6, IL-23A, CXCL1, CXCL8, MMP1 and MMP13 levels in cell culture supernatants were measured by ELISA. Date was expressed as mean±SEM(*n*=6). *n* represent biologically independent samples (A, B, E) or fields of view (C, D). The data was analyzed using two-way ANOVA. **P*＜0.05, ***P*＜0.01, ****P*＜0.001. The yellow triangle symbol marks TUNEL-positive cells.


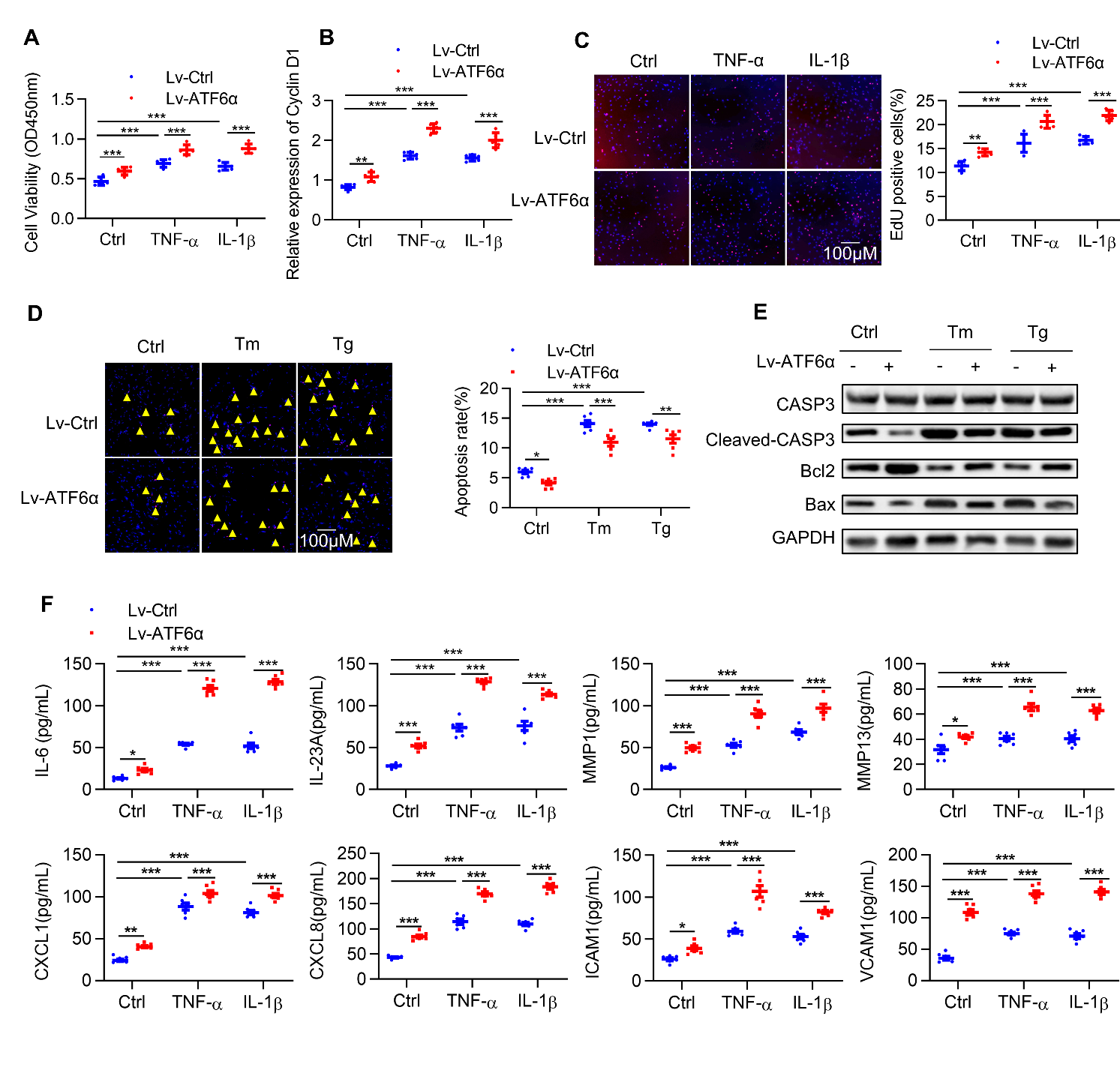


**Supplementary Figure 3**. Overexpression of ATF6α promoted proliferation and inflammation, while reduced apoptosis of OA FLSs. OA FLSs was transfected with empty lentivirus (Lv-Ctrl) or ATF6α overexpressing lentivirus (Lv-ATF6α) for 72h, followed by TNF-α (10 ng/mL) or IL-1β (10 ng/mL) for another 72 h **(A)**, 6h**(B)** or 24h **(C, F)**. **A,** Cell viability was detected via CCK-8 assay. **B,** Total cellular RNA was extracted and cyclin D1 mRNA expression was analyzed by qPCR. **C,** Cell proliferation was determined by EdU staining and EdU incorporation was calculated as EdU + cells/total cells, quantified by ImageJ. **(D, E)** OA FLSs was transfected with Lv-ATF6α and Lv-Ctrl for 72 h, followed by treatment by Tm (2μg/mL) or Tg (300 nM) for another 24 h. **D,** Apoptosis was evaluated by TUNEL assay and expressed as percentage of TUNEL-positive cells. **E,** Total protein was extracted for WB detection of CASP3, Cleaved-CAPS3, Bcl2 and Bax. **F,** Cytokine levels in cell culture supernatants were measured by ELISA. Date was expressed as mean±SD(*n*=6). *n* represent biologically independent samples (A, B, F) or fields of view (C, D). The data was analyzed using two-way ANOVA. **P*＜0.05, ***P*＜0.01, ****P*＜0.001. The yellow triangle symbol marks TUNEL-positive cells.


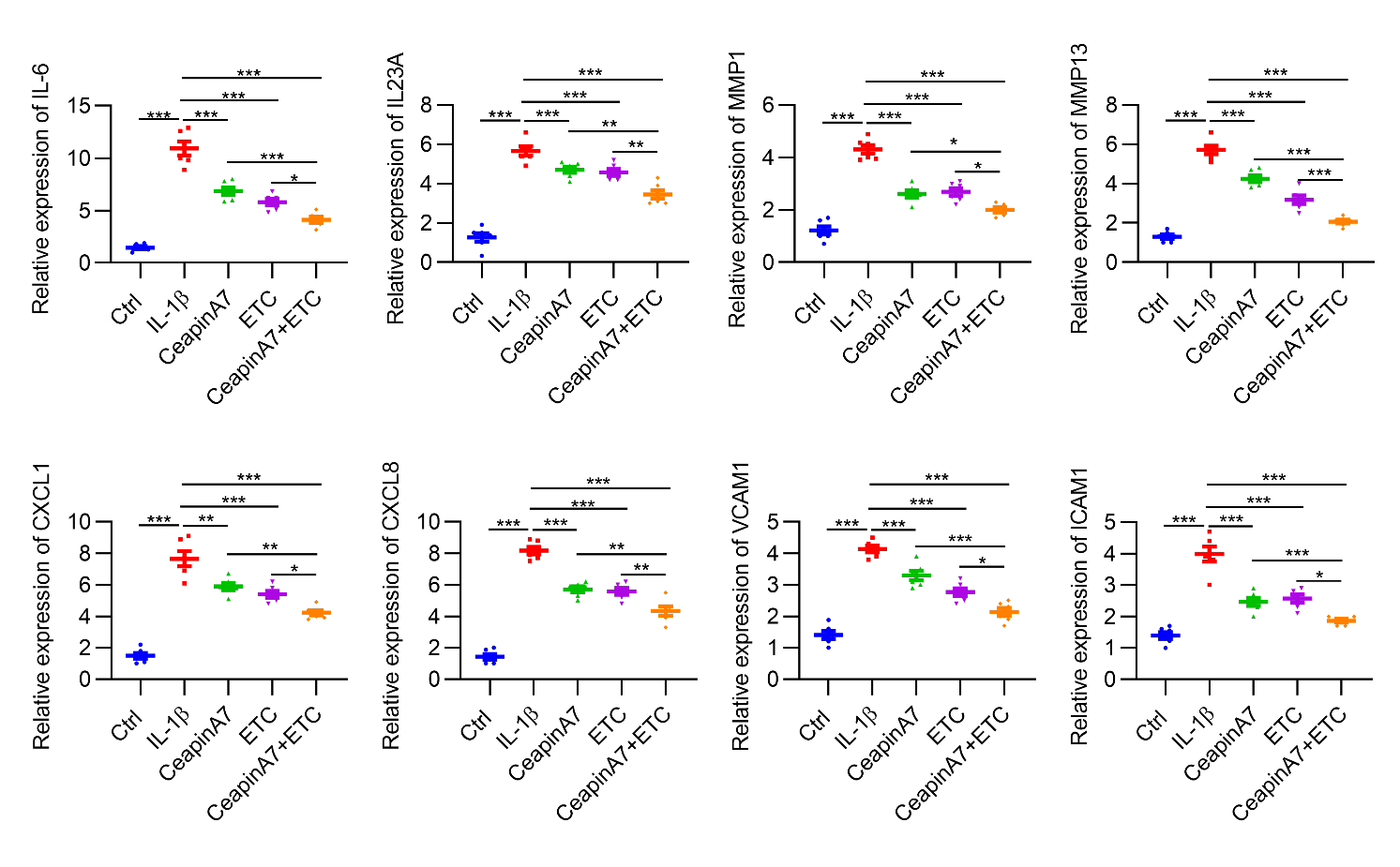


**Supplementary Figure 4**. Ceapin-A7 and ETC synergized to inhibit cytokines expression in RA FLSs. RA FLSs was pretreated with Ceapin-A7, ETC, or combination for 2h before being stimulated by IL-1β (10 ng/mL) for 24h. IL-6, IL-23A, ICAM1, VCAM1, CXCL1, CXCL8, MMP1 and MMP13 mRNA expression was analyzed by qPCR. Date was expressed as mean±SD(*n*=6). *n* represent biologically independent samples. The data was analyzed using one-way ANOVA. **P*＜0.05, ***P*＜0.01, ****P*＜0.001.


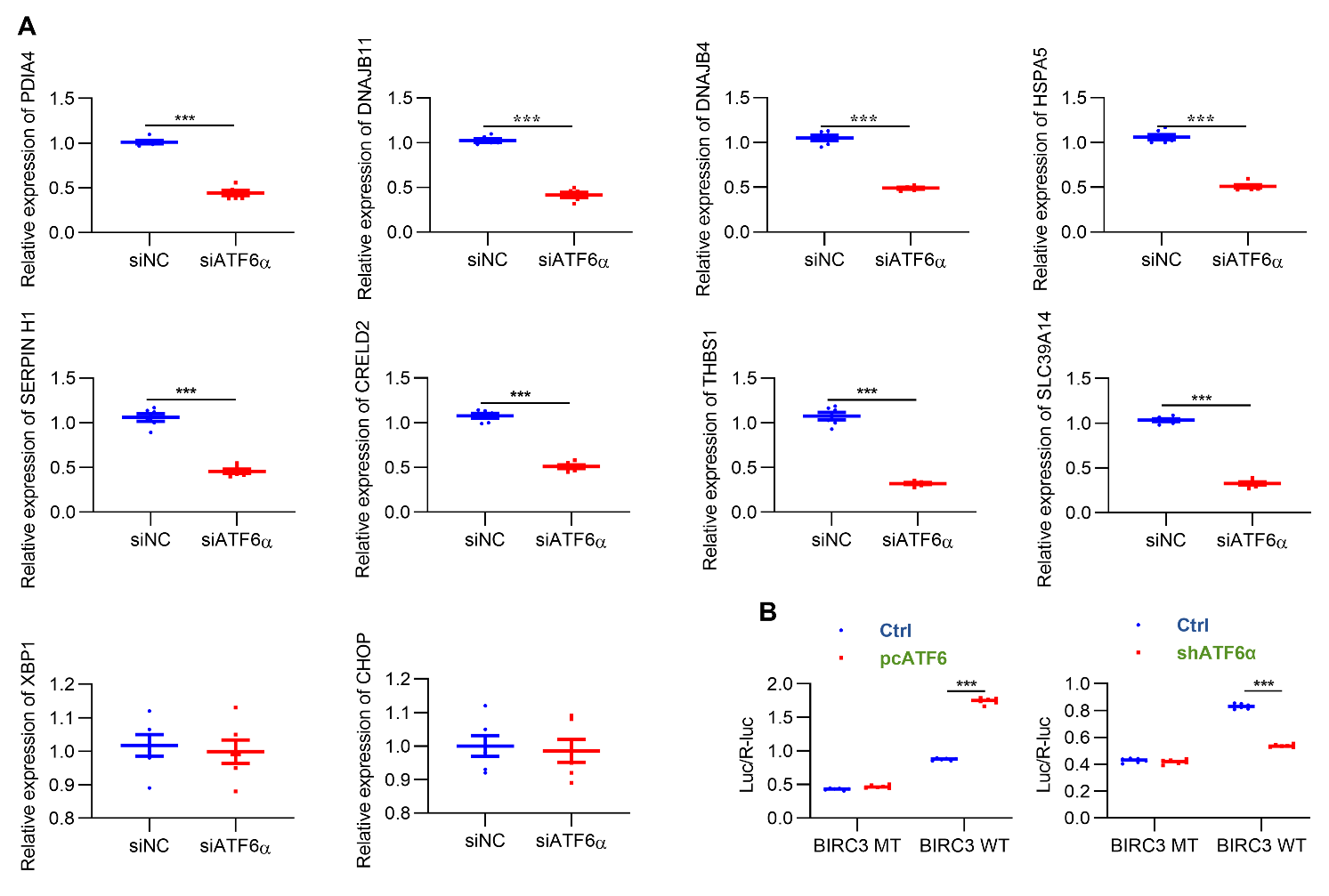
**Supplementary Figure 5.** Validation of differentially expressed genes from RNAseq and the activity of BIRC3 promoter. **A,** RA FLSs was transfected with ATF6α-siRNA for 72 h. Representative ER stress-related genes were measured by qPCR. **B,** BIRC3 promoter activity was assessed with the dual luciferase reporter assay. Date was expressed as mean±SD (*n*=6). *n* represent biologically independent samples. The data was analyzed using two- tailed unpaired Student’ s *t* test. ****P*＜0.001.

**
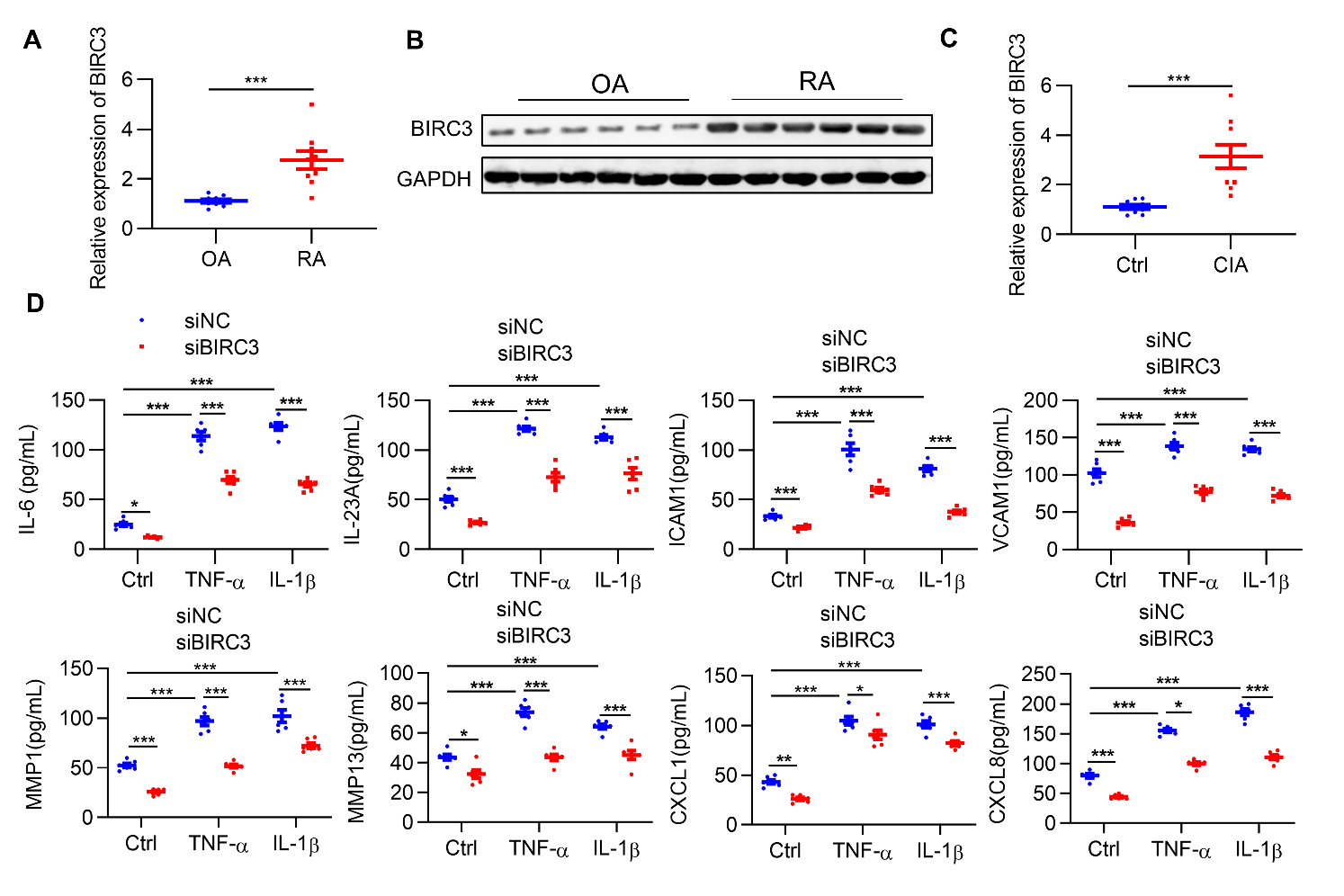
**

**Supplementary Figure 6**. BIRC3 regulates inflammation of RA FLSs. BIRC3 mRNA(**A)** and protein**(B)** expression was assessed in RA FLSs and OA FLSs (*n* = 9). **C,** BIRC3 mRNA expression was assessed by qPCR in the knee joints from normal DBA1 mice (Ctrl) and CIA mice (CIA) (*n* = 9). **D,** RA FLSs were transfected with BIRC3-siRNA for 72 h, followed by treatment with TNF-α (10 ng/mL) or IL-1β (10 ng/mL) for another 24 h. Cytokines in cell supernatants was assessed by ELISA. Data were expressed as mean ± SD and analyzed using two-tailed unpaired Student’ s *t* test **(A, C)** and multiple *t* test analysis **(D)**. *n* represent biologically independent samples (A, C, D). **P*＜0.05, ***P*＜0.01, ****P*＜0.001.

## Supplementary Tables

| Supplementary Table 1 Clinical characteristics of RA patients | | | | | | | | |
| --- | --- | --- | --- | --- | --- | --- | --- | --- |
| Sample no. | Gender | Age | Diagnosis | DAS28 | RF  (IE/ml) | CRP  (mg/L) | Anti-CCP (E/mL) | Duration (mon) |
| 1 | M | 53 | RA | 3.9 | 56 | 899 | 26 | 43 |
| 2 | M | 65 | RA | 5.3 | 67 | 788 | 59 | 101 |
| 3 | M | 59 | RA | 4.6 | 55 | 55 | 83 | 78 |
| 4 | M | 62 | RA | 5.2 | 43 | 66 | 47 | 42 |
| 5 | M | 63 | RA | 5.1 | 78 | 67 | 89 | 55 |
| 6 | M | 57 | RA | 4.8 | 98 | 128 | 556 | 19 |
| 7 | M | 58 | RA | 5.6 | 123 | 55 | 233 | 12 |
| 8 | M | 69 | RA | 4.4 | 155 | 44 | 41 | 21 |
| 9 | M | 70 | RA | 4.1 | 79 | 267 | 345 | 29 |
| 10 | M | 52 | RA | 4.9 | 201 | 321 | 807 | 33 |
| 11 | F | 53 | RA | 4.2 | 189 | 77 | 53 | 55 |
| 12 | F | 66 | RA | 4.9 | 129 | 54 | 268 | 76 |
| 13 | F | 58 | RA | 3.7 | 67 | 69 | 423 | 121 |
| 14 | F | 64 | RA | 4.1 | 88 | 81 | 890 | 87 |
| 15 | F | 69 | RA | 5.7 | 56 | 156 | 111 | 102 |
| 16 | F | 61 | RA | 5.2 | 49 | 291 | 59 | 33 |
| 17 | F | 55 | RA | 5.7 | 155 | 189 | 572 | 44 |
| 18 | F | 50 | RA | 4.8 | 107 | 233 | 489 | 134 |
| 19 | F | 54 | RA | 3.9 | 99 | 401 | 66 | 64 |
| 20 | F | 67 | RA | 4.3 | 54 | 49 | 84 | 78 |

| Supplementary Table 2 Primers used in this study | | |
| --- | --- | --- |
| Gene | Forward | Reverse |
| CHOP | TAACAGCAACAAGGAGACGA | AGAGTTGGTGCAGATGG |
| GAPDH | GCACCGTCAAGGCTGAGAAC | TGGTGAAGACGCCAGTGGA |
| ATF6α | CACAGCTCCCTAATCACGTGG | ACTGGGCTA TTCGCTGAAGG |
| ATF6α(Mus) | GTCCCTTCTCCGTCCTCTG | CGCCCACAATCGGTTTC |
| ATF6β | CAGCCATCAGCCACAACAAG | GGCATCACCAGGGACATCTT |
| BIRC3 | GTTCTCTGACCCAACCCAGA | GAGCAATTGTTGGCTGATGA |
| BIRC3(Mus) | GCCTAGCTGGGGACGATTTA | GGAAATGCCTCTGGTGCTCT |
| BIRC2 | GAGCGGGCCGTATCTCCTT | CCTGTGCCTGCTATAGTGCTT |
| PDIA4 | CACTGCAAGAAACTTGCCCC | GACGTCCATCATGTGGCTCC |
| DNAJB11 | TAGAGCCTGGGGTGAGAGAC | GAGCCCTTCCCCTTTCTTCC |
| DNAJB4 | CGTAGTTTCTCCCACCCCAT | GCCATGAAAGGTGTACCGGA |
| HSPA5 | GGAACCATCCCGTGGCATAA | CTTGGTAGGCACCACTGTGT |
| SERPINH1 | CCAGCCCGACCCAGAATGAA | TCTCATCCCAGTGTGGCTTG |
| CRELD2 | CCGGCGCCGTCAAGTAG | GGTAAGAATCGGAGAGGCCG |
| THBS1 | CCCTTGTGCTCAGAGTGGAT | GCCAGTAGAGAACAAATAAGCATGG |
| SLC39A14 | TTCACCCCTGGCATTAGCAG | CACCCGTGGGATTCTCAACA |
| IL23A | CAAAGCAAGTGGAAGTGGGC | TGAGTGCCATCCTTGAGCTG |
| XBP1 | CCCTCCAGAACATCTCCCCAT | ACATGACTGGGTCCAAGTTGT |

| Supplementary Table 3 Primers used in this study | |
| --- | --- |
| siRNA | 5’-3’ |
| ATF6α siRNA-1 | GCAACCAATTATCAGTTTA |
| ATF6α siRNA-2 | GCTTGTCAGTCTCGCAAGA |
| ATF6α siRNA-3 | CCATCATTATCAGACAGT |
| BIRC3 siRNA-1 | CCTGGATAGTCTACTAACT |
| BIRC3 siRNA-2 | CGTGGCTCTTATTCAAACT |
| BIRC3 siRNA-3 | GCGCCAACACGTTTGAACT |

| Supplementary Table 4 Chip-PCR Primers used in this study | | |
| --- | --- | --- |
| Gene | Forward | Reverse |
| BIRC2 | GTTTGTCTCCAGAACAGAAAACCA | AGCTGCCTGTGCAAATCGAA |
| BIRC3 | CCAGGCAGGCTAAGCAATGA | CTCGGGGATTTCCATGACCC |

| Supplementary Table 5 Routine blood parameters | | | | | | |
| --- | --- | --- | --- | --- | --- | --- |
| Terms | Units | Mean value±SD(*n*=9) | | | | significant difference |
|  |  | Vehicle | Ceapin-A7 | ETC | Ceapin-A7+ETC |  |
| WBC | 10^9/L | 5.9±1.9 | 5.4±1.6 | 5.1±1.3 | 4.6±1.0 | n.s |
| Lymph# | 10^9/L | 4.4±1.2 | 3.9±1.2 | 4.2±1.1 | 3±1.6 | n.s |
| Mon# | 10^9/L | 0.2±0.1 | 0.2±0.1 | 0.1±0.1 | 0.3±0.1 | n.s |
| Gran# | 10^9/L | 1.3±0.5 | 1.3±0.45 | 0.8±0.4 | 1.3±0.5 | n.s |
| Lymph% | % | 74.6±22.1 | 73±22.3 | 82.5±19.8 | 65.1±21.6 | n.s |
| Mon% | % | 2.7±0.8 | 3±0.5 | 2.7±0.6 | 3.5±0.7 | n.s |
| Gran% | % | 22.7±3.1 | 24±3.8 | 14.8±4.2 | 28.4±3.9 | n.s |
| RBC | 10^12/L | 10.74±2.7 | 9.9±2.6 | 9.76±2.1 | 9.13±1.8 | n.s |
| HGB | g/L | 171±18.9 | 152±18 | 152±20.3 | 145±19.7 | n.s |
| HCT | % | 59±16 | 53.4±17 | 54.7±16 | 51.4±19 | n.s |
| MCV | fL | 55±13 | 54±16 | 56.1±14.5 | 56.4±16.7 | n.s |
| MCH | pg | 15.9±4.8 | 15.3±4.8 | 15.5±3.6 | 15.8±4.2 | n.s |
| MCHC | g/L | 289±34 | 284±29 | 277±32 | 282±35 | n.s |
| RDW | % | 16.1±2.9 | 18.4±3.4 | 20.3±2.6 | 18.1±15.8 | n.s |
| PLT | 10^9/L | 857±334 | 1375±345 | 1423±266 | 1719±289 | n.s |
| MPV | fL | 6±2 | 5.9±2.3 | 5.9±1.9 | 6.3±2.2 | n.s |
| PDW |  | 17±0.23 | 16.7±1.1 | 16.5±0.8 | 16.9±0.7 | n.s |

| Supplementary Table 6 Serum biochemical analysis | | | | | | | |
| --- | --- | --- | --- | --- | --- | --- | --- |
| Terms | Units |  | Mean value±SD(*n*=9) | | | | significant difference |
|  |  | Vehicle | | Ceapin-A7 | ETC | Ceapin-A7+ETC |  |
| AST | U/L | 102.8±23.6 | | 123.3±16.8 | 121.6±17.8 | 108.7±5.9 | n.s |
| ALP | U/L | 80.5±9.6 | | 107.8±15.6 | 86.9±12.9 | 108.5±14.1 | n.s |
| γ-GT | U/L | 1.58±0.34 | | 1.44±0.66 | 1.29±0.44 | 1.81±0.38 | n.s |
| CHOL | mmol/L | 3.09±0.45 | | 3.11±1.12 | 2.89±0.82 | 4.23±0.77 | n.s |
| GLU | mmol/L | 8.88±2.99 | | 10.03±3.45 | 9.95±2.86 | 9.76±3.2 | n.s |
| CREA | μmol/L | 28.03±9.8 | | 28.26±8.75 | 26.67±7.88 | 28.69±9.6 | n.s |
| UA | μmol/L | 179.6±33.6 | | 158.9±22.4 | 163.9±23.6 | 171.8±27.8 | n.s |
